# Effects of aquatic exercise on pain and physical function in overweight/obese patients with lower limb osteoarthritis: a systematic review and meta-analysis

**DOI:** 10.3389/fphys.2026.1864401

**Published:** 2026-06-16

**Authors:** Wei Zhou, Yu Yan, Houjie Zhou, Zhijian Zhang, Juntao Yan

**Affiliations:** 1 College of Education, Beijing Sport University, Beijing, China; 2 Chongqing Luneng Bashu Secondary School, Chongqing, China

**Keywords:** aquatic exercise, lower limb osteoarthritis, obesity, overweight, pain, physical function

## Abstract

**Background:**

Previous research has established that aquatic exercise is an effective intervention for alleviating pain and enhancing physical function in patients with lower limb osteoarthritis (OA). However, its specific efficacy for overweight and obese populations, as well as the intervention parameters associated with clinical outcomes in these groups, remain poorly defined. This study aims to assess the effects of aquatic exercise on pain and physical function in this specific demographic and to explore potential intervention-related moderators through subgroup analysis.

**Methods:**

Following the PRISMA guidelines, a systematic search was conducted across the PubMed, Web of Science, Embase, Cochrane Library, and Scopus databases. The search cutoff date was set for February 1, 2026. Emphasis was placed on randomized controlled trials (RCTs) evaluating aquatic interventions for overweight or obese adults with primary knee and/or hip OA.

**Results:**

A total of 12 RCTs involving 1,057 participants were included. The random-effects meta-analysis suggested that aquatic exercise was associated with lower pain scores (SMD = -0.36; 95% CI, -0.50 to -0.22; *p* < 0.001; *I²* = 18%) and better physical function (SMD = -0.33; 95% CI, -0.46 to -0.20; *p* < 0.001; *I²* = 0%). Exploratory subgroup analyses suggested that both overweight and obese patients showed favorable changes in pain and physical function, with numerically larger effects observed in obese patients. Lower volume protocols, including shorter session durations, lower weekly frequency, and shorter intervention periods, tended to show favorable outcomes; however, these subgroup findings should be interpreted as hypothesis-generating rather than confirmatory evidence of an optimal dose-response relationship.

**Conclusion:**

Aquatic exercise may be associated with modest improvements in pain and physical function among overweight or obese patients with lower limb OA. Exploratory subgroup findings suggested numerically larger effects in obese individuals and favorable outcomes with some lower volume protocols. However, the certainty of evidence was low, and these findings should be interpreted cautiously. Current evidence does not support firm conclusions regarding an optimal aquatic exercise prescription or a definitive dose-response relationship. Aquatic exercise may represent a useful low-impact adjunct to conservative management, but further well-designed trials are needed to confirm clinically meaningful effects and clarify optimal intervention parameters.

**Systematic review registration:**

https://www.crd.york.ac.uk/PROSPERO/view/CRD420261354574, identifier CRD420261354574.

## 1 Introduction

Osteoarthritis (OA) is the most prevalent chronic degenerative disease globally. According to the Global Burden of Disease Study 2021, OA affects approximately 595 million people, accounting for 7.6% of the global population (Steinmetz et al., 2023). Driven by population aging and metabolic diseases, the number of cases has increased by approximately 132.2% since 1990 (Wieland et al., 2005; Steinmetz et al., 2023). Although aging is the pathogenic foundation, OA is not an inevitable consequence of biological aging; a high body mass index (BMI) has emerged as the most critical determinant (Allen et al., 2022). As a primary risk factor for lower limb OA, high BMI accounted for approximately 35.8% of OA-related disability-adjusted life years (DALYs) in 2021 (Wang et al., 2025). The years lived with disability (YLDs) attributable to high BMI have tripled globally, surging from 1.449 million in 1990 to 4.422 million in 2021 (Lu et al., 2025).

In clinical practice, there are currently no definitive disease-modifying OA drugs (DMOADs) capable of reversing disease progression or altering joint structure (Wieland et al., 2005). Existing pharmacological treatments primarily rely on pain relief, with core regimens including the use of acetaminophen, nonsteroidal anti-inflammatory drugs (NSAIDs), and intra-articular injections of corticosteroids or hyaluronic acid (Yu and Hunter, 2015; Hermann et al., 2018). However, in overweight and obese populations with lower limb OA, long-term reliance on medications for symptom relief significantly increases the incidence of peptic ulcers, acute renal failure, ischemic stroke, and myocardial infarction (Han et al., 2025), and may accelerate the degeneration of joint structures and the deterioration of joint function (Salis and Sainsbury, 2024). Furthermore, end-stage surgical options, such as total knee and hip arthroplasty, carry extremely high risks for overweight and severely obese patients, including poor wound healing, periprosthetic joint infection, deep vein thrombosis, and venous thromboembolism (Lützner et al., 2009). Consequently, authoritative international organizations and relevant studies have elevated non-pharmacological interventions to a central role (McAlindon et al., 2014; Bannuru et al., 2019). Non-pharmacological approaches focus on lifestyle modifications, particularly “exercise therapy,” to achieve clinically meaningful pain relief (Messier, 2010; Fransen et al., 2015). Despite clinical recommendations, traditional land-based exercises face substantial implementation barriers in obese populations. Patients frequently fall into a “pain-avoidance cycle,” where excess body weight places immense mechanical stress on the joints, thereby exacerbating pain during weight-bearing exercises. This can trigger Kinesio phobia, leading to a sedentary lifestyle and further muscle atrophy (Huffman et al., 2024). Furthermore, beyond intrinsic factors, multifactorial cognitive, social, and environmental factors can either facilitate or impede exercise adherence among OA patients (Giardulli et al., 2025).

Aquatic exercise provides a unique physical microenvironment that can compensate for the limitations of land-based training. Buoyancy significantly reduces static mechanical stress; standing in chest-deep water can decrease knee joint loading by 65% to 70%, thereby enabling pain-free range-of-motion training (Thein and Brody, 1998). Hydrostatic pressure optimizes cardiovascular microcirculation by promoting venous return and alleviating periarticular edema. The viscosity of water provides adaptive isokinetic resistance, which safely enhances muscle strength without increasing the risk of mechanical overload commonly associated with free-weight training (Becker, 2009). Finally, the thermodynamic properties of warm water (32 °C–34 °C) trigger neurophysiological regulations, blocking nociceptive impulses through the “gate control theory” and reducing muscle stiffness (Melzack and Katz, 2004).

Previous studies have confirmed that aquatic exercise can significantly improve the symptoms of lower limb OA. Lim et al. (2010) conducted an 8-week aquatic exercise study and observed significant improvements in both pain symptoms and physical function among participants. Similarly, Rewald et al. (2020) implemented a 12-week aquatic cycling training program for patients with knee OA, reporting effective improvements in pain symptoms and physical function. However, current research presents mixed findings. For instance, Lund et al. (2008) implemented an 8-week aquatic exercise intervention, yet at the end of the intervention, aquatic exercise did not demonstrate superior improvement effects. Discrepancies among the existing literature hinder a definitive consensus regarding aquatic exercise. This indicates that varying study outcomes are likely attributable to differences in participant characteristics and specific intervention parameters.

Although several systematic reviews have attempted to comprehensively analyze the effects of aquatic exercise on pain and physical function, numerous limitations remain. An early review by Bartels et al. (2016) confirmed the overall effectiveness of aquatic exercise but primarily focused on the general OA patient population without separately analyzing its impact on high-BMI individuals. Therefore, it remains unclear whether aquatic exercise can confer “amplified” benefits to overweight or obese populations. A 2022 study by Jurado-Castro et al. (2022) confirmed that exercise could improve function in obese patients with knee OA, but this study failed to isolate the specific efficacy of aquatic exercise. Among other reviews targeting overweight or obese populations, a review by Song and Oh (2022) explored the effects of aquatic exercise on OA symptoms but did not establish strict BMI cut-off values or perform subgroup stratification. In summary, most meta-analyses have not specifically considered the overweight and obese populations, which weakens the identification of the specific effects of aquatic exercise on this demographic. Beyond identifying the specific efficacy for this demographic, another critical limitation lies in clinical application. Translating aquatic interventions into routine clinical practice requires evidence-based exercise prescriptions to maximize therapeutic benefits while minimizing joint stress. However, few studies have deeply explored the dose-response relationship of individual training parameters (e.g., specific session duration and weekly frequency). This gap hinders the precise formulation of exercise prescriptions in clinical practice and impedes the broader application of aquatic exercise among overweight or obese patients with lower limb OA.

Therefore, this study aims to synthesize the latest RCTs to evaluate the effects of aquatic exercise on pain and physical function specifically in individuals with concurrent overweight/obesity and lower limb OA. Furthermore, through exploratory subgroup analyses, we aimed to examine whether intervention parameters, such as frequency and duration, were associated with differences in treatment effects.

## 2 Materials and methods

### 2.1 Design

This systematic review and meta-analysis was conducted in accordance with the PRISMA 2020 guidelines and the Cochrane Handbook (Higgins and Cochrane Collaboration, 2019; Page et al., 2021). with its protocol prospectively registered in PROSPERO (CRD420261354574).

### 2.2 Search strategy

Guided by the PRISMA framework, a comprehensive literature retrieval was performed from database inception to February 1, 2026. The search encompassed five databases: Scopus, Web of Science, Embase, PubMed, and the Cochrane Library. We combined MeSH headings and free-text terms such as “Aquatic exercise,” “Osteoarthritis,” and “exercise training protocol” to formulate the search syntax. To capture potentially missed records, we manually screened the reference lists of recent, highly cited systematic reviews and examined the Cochrane Central Register of Controlled Trials for unpublished data.

### 2.3 Eligibility criteria

Inclusion criteria comprise: (1) Population: Adult patients (aged ≥ 18 years) diagnosed with primary lower limb OA (specifically involving the knee and/or hip joints). Participants were required to be overweight or obese (overweight 25 ≤ BMI < 30 kg/m² or obese BMI ≥ 30 kg/m²) and had not engaged in regular or strenuous physical activity prior to the trial. (2) Intervention: Aquatic exercise. (3) Comparison: No intervention, usual care, or a non-aquatic intervention (e.g., land-based exercise, home-based exercise, or passive balneotherapy). (4) Outcomes: Availability of OA-related outcome measures, such as pain scores and subjective physical function. (5) Study design: Randomized controlled trials (RCTs).

Studies were excluded if they met any of the following criteria: (1) non-English publications; (2) non-primary research such as review articles, case reports, observational studies, and conference abstracts; (3) animal, *in vitro* studies, or trials involving healthy populations without lower limb OA; (4) publications without full-text availability; (5) studies where outcome data could not be properly extracted or quantified using mean and standard deviation values.

### 2.4 Data extraction

Data acquisition was conducted independently by two investigators (W.Z. and Y.Y.). and any discrepancies were resolved through discussion or, if necessary, by consulting a third investigator (J.Y.). The extracted information encompassed several key dimensions: (1) basic study identifiers, such as the primary author’s name, year of publication, and total sample size; (2) specific intervention parameters, including the type, duration, frequency, and length of sessions; (3) participant characteristics (e.g., age, BMI, and health status); and (4) therapeutic outcomes and treatment effects. When standard deviations were unavailable, we calculated them from standard errors, confidence intervals (CIs), t or p values. For unreported data, we made at least three email attempts to contact corresponding authors. Graphical data were extracted using GetData Graph Digitizer (v2.20) when numerical results were only presented in figure format.

### 2.5 Methodological quality assessment

To gauge the methodological rigor of the eligible RCTs, we applied the Cochrane Risk of Bias tool version 1 (RoB 1). This instrument evaluates potential flaws across seven distinct criteria, including sequence generation, allocation concealment, blinding procedures, and selective reporting (Higgins et al., 2011; Higgins and Cochrane Collaboration, 2019). Two authors (W.Z., Y.Y.) independently assigned a judgment of “low,” “high,” or “unclear” risk to each specific domain. A third reviewer (J.Y.) was consulted to resolve any scoring discrepancies through discussion.

### 2.6 Certainty of evidence

The certainty of the synthesized evidence for both pain and physical function outcomes was evaluated utilizing the Grading of Recommendations Assessment, Development and Evaluation (GRADE) framework. Given that all included data originated from RCTS, the initial evidence quality was assigned a “high” baseline rating. Two independent reviewers (W.Z. and Y.Y.) subsequently evaluated the evidence and downgraded the certainty levels when appropriate, based on five standard domains: risk of bias, inconsistency, indirectness, imprecision, and publication bias. Any disagreements during this process were resolved through discussion with a third adjudicator (J.Y.).

The specific criteria for downgrading the evidence were pre-defined as follows (1) Risk of Bias: The rating was lowered by one or more levels if the majority of the contributing studies were judged to have a high risk of bias or if there were significant concerns regarding the randomization process, deviations from intended interventions, or missing outcome data. (2) Inconsistency: Evidence was downgraded if substantial statistical heterogeneity was detected via the *I^2^* statistic or if there were clinically significant variations in the direction of effects across studies. (3) Indirectness: The certainty was reduced if the participant populations, intervention protocols, or outcome measures in the included studies did not directly align with the specific clinical question or the PICO criteria of this review. (4) Imprecision: Downgrading occurred if the pooled 95% confidence intervals were wide, crossed the threshold for clinically meaningful benefit or harm, or if the optimal information size was not met due to an insufficient total sample size. (5) Publication Bias: The rating was lowered if evidence of small-study effects was detected through visual inspection of funnel plots or identified via significant results from Egger’s linear regression test.

### 2.7 Statistical analysis

Regarding data synthesis and effect measures, as the primary continuous outcomes (pain scores and physical function) were evaluated using various measurement scales across the included studies, the Standardized Mean Difference (SMD) was employed as the primary summary statistic. To ensure consistency in the direction of effects across different assessment tools, the mean values of scales where higher scores indicate better clinical outcomes were multiplied by -1 prior to data synthesis. This transformation ensured that all pooled data uniformly reflected the same effect direction (i.e., higher scores representing worse symptoms or poorer physical function). Pooled effect sizes and their corresponding 95% Confidence Intervals (CIs) were calculated based on post-intervention means and standard deviations extracted from individual study reports. This approach ensures that treatment effects are comparable across different assessment tools and facilitates a robust estimation of the intervention’s overall impact on both pain and functional recovery.

Statistical heterogeneity was assessed using the *I²* statistic and the chi-square test. In anticipation of potential clinical and methodological heterogeneity across the trials, such as variations in participant characteristics and intervention protocols, a random-effects model was used for all meta-analyses. Furthermore, to ensure the reliability and stability of the pooled estimates, leave-one-out sensitivity analyses were conducted to determine if the overall conclusions were disproportionately influenced by any single study.

To further investigate potential clinical and methodological moderators, exploratory subgroup analyses were performed regardless of the initial heterogeneity magnitude. These analyses focused on participant characteristics, such as BMI status categorized as overweight or obesity, as well as intervention protocol elements including session duration, weekly frequency, weekly total duration, and the total length of the intervention period. Potential publication bias was evaluated through the visual inspection of funnel plots and further quantified using Egger’s linear regression test, with all statistical procedures executed using Revman 5.4, Stata version 17.0, with a *p*-value < 0.05 considered statistically significant.

## 3 Results

### 3.1 Studies selection

**Figure 1:**
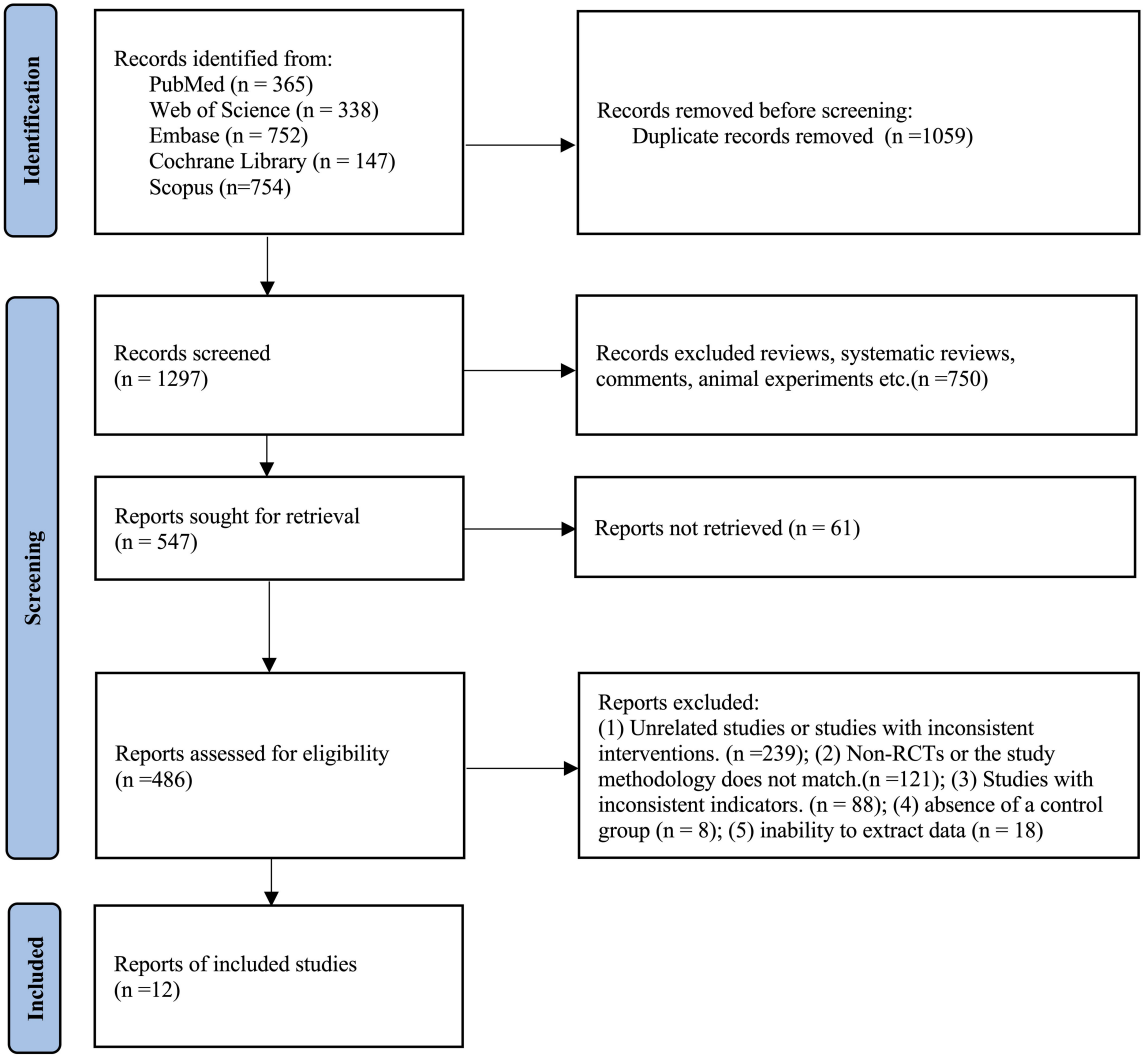
PRISMA flowchart of study selection.

As illustrated in **Figure 1**, a total of 2,356 records were initially retrieved from five electronic databases, including PubMed (n = 365), Web of Science (n = 338), Embase (n = 752), Cochrane Library (n = 147), and Scopus (n = 754). After removing 1,059 duplicate records, 1,297 studies were retained for screening. Following a preliminary review of titles and abstracts, 750 studies were eliminated because they were reviews, systematic reviews, comments, animal experiments, or other irrelevant types.

Subsequently, 547 reports were sought for retrieval, of which the full texts for 61 reports could not be obtained. The full texts of the remaining 486 reports were then assessed for eligibility. Among these, 474 reports were excluded for the following reasons: (1) unrelated studies or inconsistent interventions (n = 239); (2) non-RCTs or incompatible study methodologies (n = 121); (3) inconsistent outcome indicators (n = 88); (4) absence of a control group (n = 8); and (5) inability to extract data (n = 18). Ultimately, 12 studies (Regazzo et al., 2026; Cochrane et al., 2005; Fransen et al., 2007; Hinman et al., 2007; Lim et al., 2010; Wang et al., 2011; Alkatan et al., 2016; Dias et al., 2017; Waller et al., 2017; Kunduracilar et al., 2018; Kuptniratsaikul et al., 2019; Rewald et al., 2020) were included in this systematic review and meta-analysis.

### 3.2 Characteristics of the included studies

The main characteristics of the included studies are summarized in **Tables 1**, **2**. As detailed in **Table 1**, the 12 included RCTs were published between 2005 (Cochrane et al., 2005) and 2026 (Regazzo et al., 2026). The cumulative sample size was 1,057, comprising 540 participants in the intervention groups and 517 in the control groups. Across the individual trials, cohort sizes ranged from 48 (Alkatan et al., 2016) to 312 (Cochrane et al., 2005), with participants’ ages falling between 58 and 71 years. The analysis incorporated both single-gender (3 female-only studies, n=212) (Dias et al., 2017; Waller et al., 2017; Kunduracilar et al., 2018) and mixed-gender (9 studies, n=845) reports (Regazzo et al., 2026; Cochrane et al., 2005; Fransen et al., 2007; Hinman et al., 2007; Lim et al., 2010; Wang et al., 2011; Alkatan et al., 2016; Kuptniratsaikul et al., 2019; Rewald et al., 2020). In terms of clinical focus, 8 RCTs exclusively recruited patients with knee OA (Regazzo et al., 2026; Lim et al., 2010; Wang et al., 2011; Dias et al., 2017; Waller et al., 2017; Kunduracilar et al., 2018; Kuptniratsaikul et al., 2019; Rewald et al., 2020), whereas 4 RCTs involved subjects with lower limb OA (hip and knee) (Cochrane et al., 2005; Fransen et al., 2007; Hinman et al., 2007; Alkatan et al., 2016).

**Table 1:** Characteristics of the studies included in this meta-analysis (Participants’ characteristics).

Author/year	Country /region	Sample size(n)	Gender male/female	Adherence	Mean age(y)	Mean BMI (kg/m2)	Participants	Clinical focus
Alkatan 2016	USA	Int:24 Con:24	2/22 2/22	98%	59 ± 2 61 ± 1	34.6 ± 10.29 31.6 ± 8.33	Obesity	Knee and Hip
Cochrane 2005	UK	Int:153 Con:159	56/97 60/99	53.5% (1-year main trial)	69.6 ± 6.8 69.63 ± 6.26	29.7 ± 5.1 29.8 ± 5.1	Overweight	Knee and Hip
Dias 2017	Brazil	Int:33 Con:33	0/33 0/33	83.3–100% 30/33 attended all sessions	70.8 ± 5 71 ± 5.2	30.5 ± 4.3 30 ± 5.2	Obesity	Knee
Fransen 2007	Australia	Int:55 Con:41	15/40 7/34	81% attended ≥50% of sessions	70 ± 6.3 69.6 ± 6.1	30 ± 5 30.7 ± 5	Obesity	Knee and Hip
Hinman 2007	Australia	Int:36 Con:35	12/24 11/24	94% attended ≥10/12 sessions	63.3 ± 9.5 61.5 ± 7.8	33.8 ± 6.5 32.9 ± 6.6	Obesity	Knee and Hip
Kunduracilar 2018	Turkey	Int:30 Con:29	0/30 0/29	NR	63.2 ± 7.6 58.23 ± 7.55	30.9 ± 4.3 31.2 ± 6.2	Obesity	Knee
Kuptniratsaikul 2019	Thailand	Int:40 Con:40	2/38 3/37	NR	62.1 ± 6.4 61.7 ± 6.9	28.9 ± 3.2 28.4 ± 3	Overweight	Knee
Lim 2010	South Korean	Int:26 Con:24	3/23 3/21	NR	65.7 ± 8.9 63.3 ± 5.3	27.8 ± 1.6 27.9 ± 2	Overweight	Knee
Regazzo 2026	Italy	Int:30 Con:29	11/19 8/21	NR	66 ± 14 68 ± 9	27 ± 6.7 27.5 ± 8.6	Overweight	Knee
Rewald 2020	Netherlands	Int:55 Con:47	16/39 23/24	80%	59 ± 9.5 61 ± 7.4	29 ± 5.6 29 ± 5.4	Overweight	Knee
Waller 2017	Finland	Int:43 Con:44	0/43 0/44	88%	63.8 ± 2.4 63.9 ± 2.4	26.6 ± 3.8 27.1 ± 3.5	Overweight	Knee
Wang 2011	Taiwan	Int:26 Con:26	4/22 4/22	86.4%	66.7 ± 5.6 67.9 ± 5.9	26.6 ± 2.5 26.6 ± 2.08	Overweight	Knee

Int, intervention group; Con, control/comparator group; BMI, body mass index; NR, not reported.

**Table 2:** Characteristics of the studies included in this meta-analysis (Intervention characteristics).

Author/Year	Intervention	Intensity	Water temperature	Duration (Weeks)	Frequency (times/week)	Minutes per session(min)	Minutes per week (min)	Supervision	Outcomes
Alkatan 2016	SW LBE	60–70% HRR	27-28 °C	12	3	40-45	120-135	Yes	WOMAC-P, WOMAC-PF
Cochrane 2005	AQE UC	40–60% MVC/HRmax	29 °C	52	2	60	120	Yes	WOMAC-P, WOMAC-PF
Dias 2017	AQE EDU	Borg-monitored	~32 °C	6	2	40	80	Yes	WOMAC-P, WOMAC-PF
Fransen 2007	AQE WL	NR	34 °C	12	2	60	120	Yes	WOMAC-P, WOMAC-PF
Hinman 2007	AQE UC	NR	34 °C	6	2	45-60	90-120	Yes	WOMAC-P, WOMAC-PF
Kunduracilar 2018	AQE CT	NR	37-39 °C	3	5	45-60	225-300	Yes	WOMAC-P, WOMAC-PF
Kuptniratsaikul 2019	UTM HE	NR	NR	4	3	30	90	Yes	NRS
Lim 2010	AQE LBE	>65% HRmax	34 °C	8	3	40	120	Yes	BPI, WOMAC-PF
Regazzo 2026	AQE+BT BT	NR	36-38 °C	2	6	40	240	Yes	NRS, LAI
Rewald 2020	AC UC	Borg RPE 12, 50% HRmax	32 °C	12	2	45	90	Yes	KOOS-P, KOOS-ADL
Waller 2017	AQE UC	RPE 13.7–15.0, 84–85% HRmax	NR	16	3	60	180	Yes	KOOS-P, KOOS-ADL
Wang 2011	AQE UC	Borg CR10 scale 3–4	30 °C	12	3	60	180	Yes	KOOS-P, KOOS-ADL

SW, Swimming; LBE, Land-Based Exercise; AQE, Aquatic Exercise; UC, Usual Care; EDU, Educational Protocol; WL, Waiting List; CT, Conventional Therapy; UTM, Underwater Treadmill; HE, Home Exercise; BT, Balneotherapy; AC, Aquatic Cycling; HRR, heart rate reserve; MVC, maximal voluntary contraction; HRmax, maximal heart rate; RPE, rating of perceived exertion; NR, not reported; WOMAC-P/-PF, Western Ontario and McMaster Universities Osteoarthritis Index Pain subscale/Physical Function subscale; NRS, Numeric Rating Scale; LAI, Lequesne’s Algofunctional Index, BPI, Brief Pain Inventory; KOOS-P/-ADL, Knee Injury and Osteoarthritis Outcome Score Pain subscale/Physical Function subscale/Activities of Daily Living subscale.

As detailed in **Table 2**, Among the 12 included RCTs, all provided data on pain scores, assessed via tools such as the WOMAC-Pain (n = 6 (Cochrane et al., 2005; Fransen et al., 2007; Hinman et al., 2007; Alkatan et al., 2016; Dias et al., 2017; Kunduracilar et al., 2018)), KOOS-Pain (n = 3 (Wang et al., 2011; Waller et al., 2017; Rewald et al., 2020)), NRS (n = 2 (Regazzo et al., 2026; Kuptniratsaikul et al., 2019)), and BPI (n = 1 (Lim et al., 2010)). Meanwhile, 11 RCTs reported data on subjective physical function, measured by instruments like the WOMAC-Physical function (n = 7 (Cochrane et al., 2005; Fransen et al., 2007; Hinman et al., 2007; Lim et al., 2010; Alkatan et al., 2016; Dias et al., 2017; Kunduracilar et al., 2018)), KOOS-ADL (n = 3 (Wang et al., 2011; Waller et al., 2017; Rewald et al., 2020)), and LAI (n = 1 (Regazzo et al., 2026). The aquatic exercise modalities across these RCTs were as follows: 8 RCTs (Cochrane et al., 2005; Fransen et al., 2007; Hinman et al., 2007; Lim et al., 2010; Wang et al., 2011; Dias et al., 2017; Waller et al., 2017; Kunduracilar et al., 2018) employed traditional aquatic exercises, 1 RCT (Alkatan et al., 2016) employed swimming training, 1 RCT (Kuptniratsaikul et al., 2019) employed underwater treadmill exercise (UTM), 1 RCT (Rewald et al., 2020) employed aquatic cycling, and 1 RCT (Regazzo et al., 2026) employed combined balneotherapy and aquatic exercise. The intervention durations varied, ranging from a minimum of 2 weeks (Regazzo et al., 2026) to a maximum of 52 (Cochrane et al., 2005) weeks, with frequencies ranging from a minimum of 2 sessions per week (Cochrane et al., 2005; Fransen et al., 2007; Hinman et al., 2007; Dias et al., 2017; Rewald et al., 2020) to a maximum of 6 sessions per week (Regazzo et al., 2026). Additionally, we calculated the weekly exercise time based on frequency and intervention duration, with the time range spanning from 80 minutes (Dias et al., 2017) to 300 minutes (Kunduracilar et al., 2018).

Adverse event assessment and reporting varied across the included trials and are summarized in Supplementary Material **Supplementary Table 2**. Most reported adverse events were mild or transient, including joint discomfort, muscle pain, dizziness, cramps, temporary symptom exacerbation, or minor bruising (Cochrane et al., 2005; Fransen et al., 2007; Hinman et al., 2007; Lim et al., 2010; Wang et al., 2011; Alkatan et al., 2016; Dias et al., 2017; Waller et al., 2017; Kuptniratsaikul et al., 2019). However, two studies did not report adverse event information (Regazzo et al., 2026; Kunduracilar et al., 2018). One study reported a serious event involving hyperventilation at the end of a training session; the participant was hospitalized overnight, discharged the following day, and resumed training after two weeks of rest (Rewald et al., 2020). Overall, no serious adverse events were reported as intervention related.

### 3.3 Main effect

**Figure 2:**
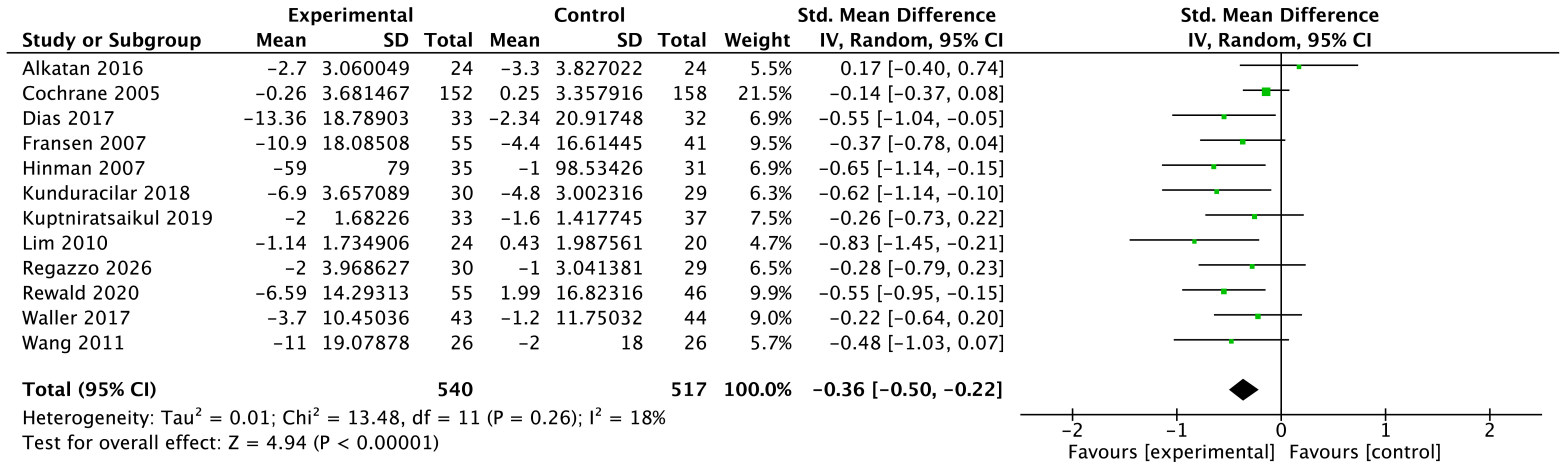
Results of the meta-analysis of the effect of aquatic exercise on pain scores in overweight and obese patients with lower limb OA.

**Figure 3:**
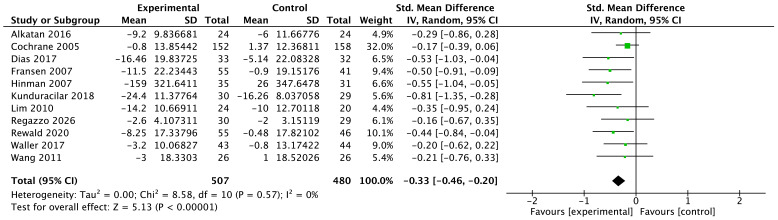
Results of the meta-analysis of the effect of aquatic exercise on physical function in overweight and obese patients with lower limb OA.

Meta-analysis results showed that the experimental intervention was associated with statistically significant improvements in both pain and physical function compared with control conditions. Specifically, the pooled estimates indicated a small-to-moderate reduction in pain scores (SMD = -0.36; 95% CI: -0.50 to -0.22; *p* < 0.001; *I²* = 18%; **Figure 2**) and a small-to-moderate improvement in physical function (SMD = -0.33; 95% CI: -0.46 to -0.20; *p* < 0.001; *I²* = 0%; **Figure 3**).

Given these findings, the pooled effects suggest small-to-moderate, statistically significant benefits favoring the experimental intervention. However, given the modest magnitude of the SMD and the clinical heterogeneity across the included studies, the clinical relevance of these findings should be interpreted cautiously. To further examine whether these results were influenced by any single study, sensitivity analyses were conducted; additionally, exploratory subgroup analyses were performed to evaluate whether the intervention effects varied across different clinical contexts.

### 3.4 Subgroup analysis

#### 3.4.1 Pain scores outcome

Subgroup analysis results for pain scores are summarized in **Table 3**. The pain-reducing effects of the intervention were consistently observed across various subgroups, encompassing participant characteristics, weekly frequency, weekly duration, session duration, and total intervention weeks.

**Table 3:** Subgroup analysis results for pain scores (Random-effects model).

Subgroup	K(n)	SMD (95% CI)	Pd	Pm	I2
Population				P = 0.50	
overweight	7(723)	-0.31 [-0.47, -0.15]	P = 0.0002		11%
obesity	5(334)	-0.42 [-0.68, -0.15]	P = 0.002		31%
Duration				P = 0.08	
>8	6(694)	-0.26 [-0.43, -0.08]	P = 0.004		18%
≤8	6(363)	-0.51 [-0.72, -0.30]	P < 0.00001		0%
Frequency				P = 0.76	
≤2	5(638)	-0.39 [-0.60, -0.18]	P = 0.0003		35%
>2	7(419)	-0.34 [-0.56, -0.12]	P = 0.002		18%
Minutes per session				P = 0.50	
<45	4(238)	-0.44 [-0.70, -0.18]	P = 0.0008		0%
≥45	8(819)	-0.33 [-0.51, -0.16]	P = 0.0002		30%
Minutes per week				P = 0.46	
≤120	7 (752)	-0.40 [-0.59, -0.22]	P < 0.0001		31%
>120	5 (305)	-0.29 [-0.53, -0.04]	P = 0.02		13%
Control Group Type				P = 0.85	
exercise comparator	3(162)	-0.29 [-0.82, 0.23]	P = 0.28		63%
non-exercise comparator	9(895)	-0.34[-0.48, -0.21]	P < 00001		0%

*K*(n), the number of studies included in the pooled effect analysis (total number of participants across pooled studies); *P*d, the *P*-value used to assess differences in effect size between subgroups; *P*m, the *P*-value for the heterogeneity test.

Regarding participant characteristics, significant pain reductions were observed in both participants with obesity (SMD = -0.42; 95% CI: -0.68 to -0.15; *p* = 0.002; *I²* = 31%) and those with overweight (SMD = -0.31; 95% CI: -0.47 to -0.15; *p* = 0.0002; *I²* = 11%), with no statistically significant difference between the two subgroups (Test for subgroup differences: *p* = 0.50). When examining intervention intensity and volume, some numerical variations in effect sizes were noted; however, subgroup differences did not reach statistical significance. For instance, interventions with a session duration of less than 45 minutes (SMD = -0.44; 95% CI: -0.70 to -0.18; *p* = 0.0008; *I²* = 0%) and those with a weekly frequency of 2 sessions or fewer (SMD = -0.39; 95% CI: -0.60 to -0.18; *p* = 0.0003; *I²* = 35%) demonstrated significant within-group improvements. Nevertheless, statistical tests indicated no significant superiority over sessions lasting ≥ 45 minutes (*p* = 0.50) or more frequent weekly sessions (*p* = 0.76).

Consistent with these trends, the analysis of intervention timing revealed that a weekly duration of 120 minutes or less resulted in significant pain reduction (SMD = -0.40; 95% CI: -0.59 to -0.22; *p* < 0.001; *I²* = 31%) but did not significantly differ from durations >120 minutes (*p* = 0.46). Additionally, a total intervention period of 8 weeks or fewer yielded a numerically larger effect size (SMD = -0.51; 95% CI: -0.72 to -0.30; *p* < 0.001; *I²* = 0%) compared to > 8 weeks (SMD = -0.26; 95% CI: -0.43 to -0.08), though the test for subgroup differences only approached statistical significance (P = 0.08). Finally, regarding the control group type, interventions compared against non-exercise comparators showed a significant pain reduction (SMD = -0.34; 95% CI: -0.48 to -0.21; *p* < 0.001; *I²* = 0%), whereas those compared against exercise comparators did not reach statistical significance within the subgroup (SMD = -0.29; 95% CI: -0.82 to 0.23; *p* = 0.28; *I²* = 63%). Nevertheless, the test for subgroup differences indicated no significant variance between the two control types (*p* = 0.85). Consequently, while certain intervention parameters generated nominally larger effect sizes, the lack of significant subgroup interactions suggests that these findings should be interpreted cautiously and not viewed as definitive evidence of optimal intervention dosages.

#### 3.4.2 Physical function outcome

Subgroup analysis results for physical function are summarized in **Table 4**. Favorable effects on physical function were observed across several subgroups, including participant characteristics, weekly frequency, weekly duration, session duration, total intervention weeks, and control group types.

**Table 4:** Subgroup analysis results for physical function (Random-effects model).

Subgroup	K(n)	SMD (95% CI)	Pd	Pm	I2
Population				P = 0.02	
overweight	6 (653)	-0.23 [-0.38, -0.07]	P = 0.004		0%
obesity	5 (334)	-0.54 [-0.76, -0.32]	P < 0.00001		0%
Duration				P = 0.12	
>8	6 (694)	-0.27 [-0.42, -0.12]	P = 0.0005		0%
≤8	5 (293)	-0.49 [-0.72, -0.25]	P < 0.0001		0%
Frequency				P = 0.86	
≤2	5 (638)	-0.35 [-0.52, -0.18]	P < 0.0001		10%
>2	6 (349)	-0.33 [-0.54, -0.11]	P = 0.003		0%
Minutes per session				P = 0.92	
<45	3 (168)	-0.35 [-0.66, -0.05]	P = 0.02		0%
≥45	8 (819)	-0.34 [-0.48, -0.19]	P < 0.00001		7%
Minutes per week				P = 0.93	
≤120	6 (682)	-0.33 [-0.49, -0.18]	P < 0.0001		0%
>120	5 (305)	-0.32 [-0.55, -0.09]	P = 0.006		3%
Control Group Type				P = 0.93	
exercise comparator	2(92)	-0.32 [-0.73, 0.09]	P = 0.13		0%
non-exercise comparator	9(895)	-0.34 [-0.48, -0.20]	P < 0.00001		7%

*K*(n), the number of studies included in the pooled effect analysis (total number of participants across pooled studies); *P*d, the *P*-value used to assess differences in effect size between subgroups; *P*m, the *P*-value for the heterogeneity test.

Regarding participant characteristics, improvements in physical function were observed in both participants with obesity (SMD = -0.54; 95% CI: -0.76 to -0.32; *p* < 0.001; *I²* = 0%) and those with overweight (SMD = -0.23; 95% CI: -0.38 to -0.07; *p* = 0.004; *I²* = 0%). The test for subgroup differences indicated a larger effect in the obesity subgroup than in the overweight subgroup (*p* = 0.02).

When examining intervention volume and timing, within-subgroup effects were generally favorable, but tests for subgroup differences did not indicate clear between-subgroup differences. For instance, interventions with a session duration of < 45 minutes (SMD = -0.35; 95% CI: -0.66 to -0.05; *p* = 0.02; *I²* = 0%) and ≥ 45 minutes (SMD = -0.34; 95% CI: -0.48 to -0.19; *p* < 0.001; *I²* = 7%) showed similar effect estimates, with no evidence of a between-subgroup difference (*p* = 0.92). Similarly, no significant subgroup differences were found for weekly frequency (≤ 2 sessions vs. >2 sessions, *p* = 0.86) or weekly duration (≤ 120 minutes vs. > 120 minutes, p = 0.93). A total intervention period of ≤ 8 weeks yielded a numerically larger effect size (SMD = -0.49; 95% CI: -0.72 to -0.25; *p* < 0.001; *I²* = 0%) compared with > 8 weeks (SMD = -0.27; 95% CI: -0.42 to -0.12; *p* = 0.0005; *I²* = 0%); however, this difference was not statistically significant (*p* = 0.12). Regarding control group type, interventions compared with non-exercise comparators showed improvement in physical function (SMD = -0.34; 95% CI: -0.48 to -0.20; *p* < 0.001; *I²* = 7%), whereas the effect in trials with exercise comparators was less precise and did not reach statistical significance (SMD = -0.32; 95% CI: -0.73 to 0.09; *p* = 0.13; *I²* = 0%). Nevertheless, the test for subgroup differences indicated no significant difference between the two comparator types (*p* = 0.93).

Overall, statistical heterogeneity was low across most subgroups. The subgroup analysis suggested a larger functional effect in participants with obesity, whereas no clear subgroup differences were identified across specific protocol parameters or comparator types. Therefore, the current data do not support firm conclusions regarding an optimal aquatic exercise dose, although favorable functional outcomes were observed across several intervention contexts.

### 3.5 Risk of bias

**Figure 4:**
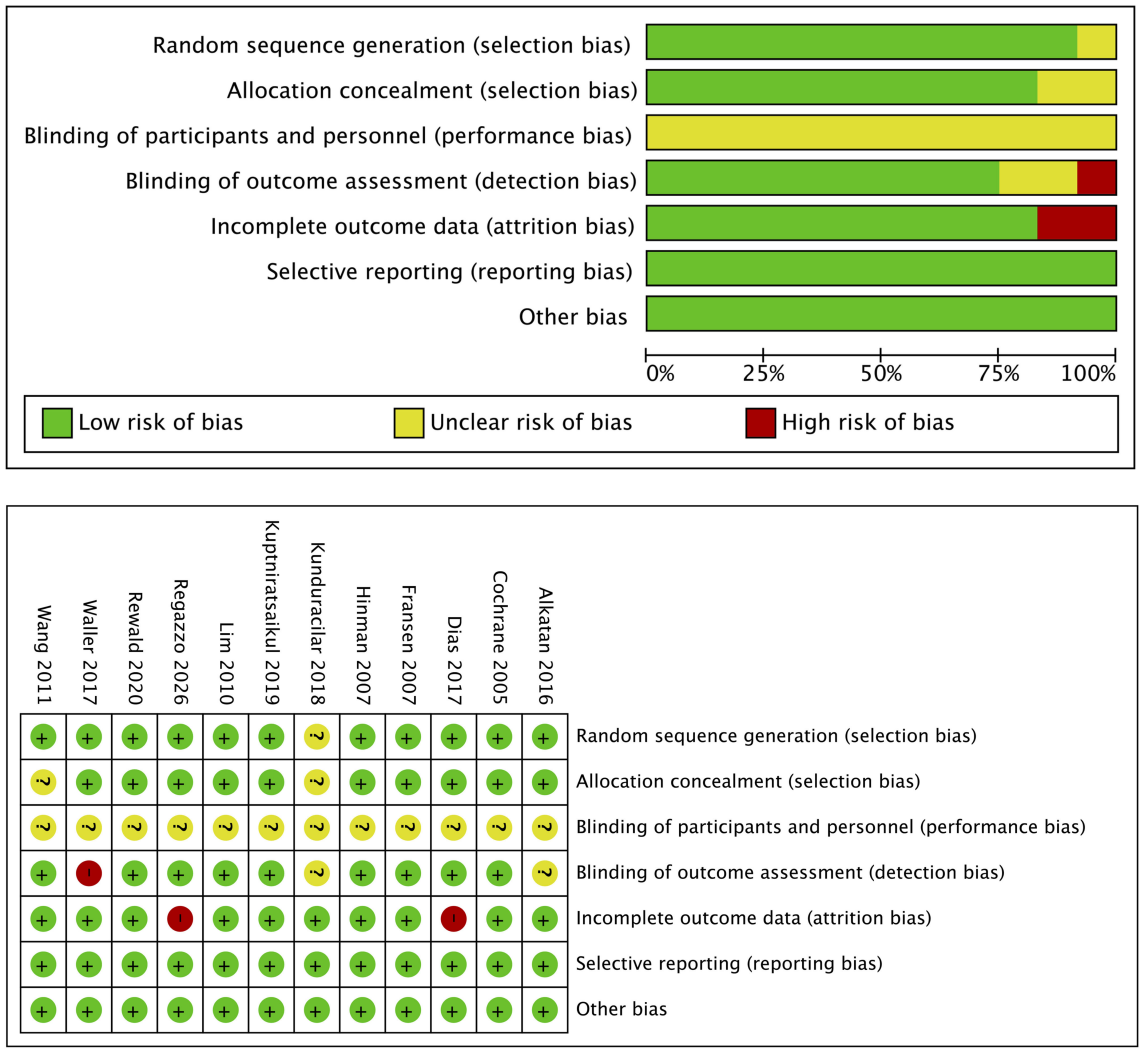
Risk assessment of included studies.

The methodological quality of the included trials was assessed using the Cochrane Risk of Bias tool, and the results are summarized in **Figure 4**. Most studies demonstrated a low risk of bias regarding random sequence generation, allocation concealment, and selective reporting. Detection bias was generally low, although a small proportion of trials lacked adequate blinding for outcome assessment. The primary methodological limitations across the included studies were performance bias and attrition bias. Due to the inherent nature of physical training interventions, blinding of participants and personnel is generally unfeasible, resulting in a widespread risk of performance bias. Furthermore, incomplete outcome data introduced a high risk of attrition bias in several studies. Because these biases can systematically overestimate the observed intervention effects, the overall pooled results should be interpreted with caution, despite the adequate trial design in other domains.

### 3.6 GRADE evaluation of evidence quality

Using the GRADE framework, we evaluated the certainty of evidence for the intervention’s effect on pain scores and physical function (**Table 5**). While all data were derived from RCTs initially categorized as high-quality evidence, the final certainty rating for both outcomes was downgraded to ‘low’. The evidence was downgraded by one level for risk of bias due to the widespread lack of blinding (performance bias) across the included trials. Additionally, despite low statistical heterogeneity, the evidence was downgraded an additional level for inconsistency to account for the substantial clinical heterogeneity inherent in the varied physical training protocols and participant characteristics. Other domains, including indirectness, imprecision, and publication bias, did not warrant further downgrading.

**Table 5:** GRADE assessment of primary outcomes.

Outcome	No of participants (studies)	Risk of bias	Inconsistency	Indirectness	Imprecision	Other considerations	SMD (95% CI)	GRADE*
Pain scores	1057(12)	Serious	Serious	Not serious	Not serious	Not serious	-0.36 (-0.50, -0.22)	⊕⊕⚪⚪ Low
Physical Function	987(11)	Serious	Serious	Not serious	Not serious	Not serious	-0.33 (-0.46, -0.20)	⊕⊕⚪⚪ Low

### 3.7 Publication bias

**Figure 5:**
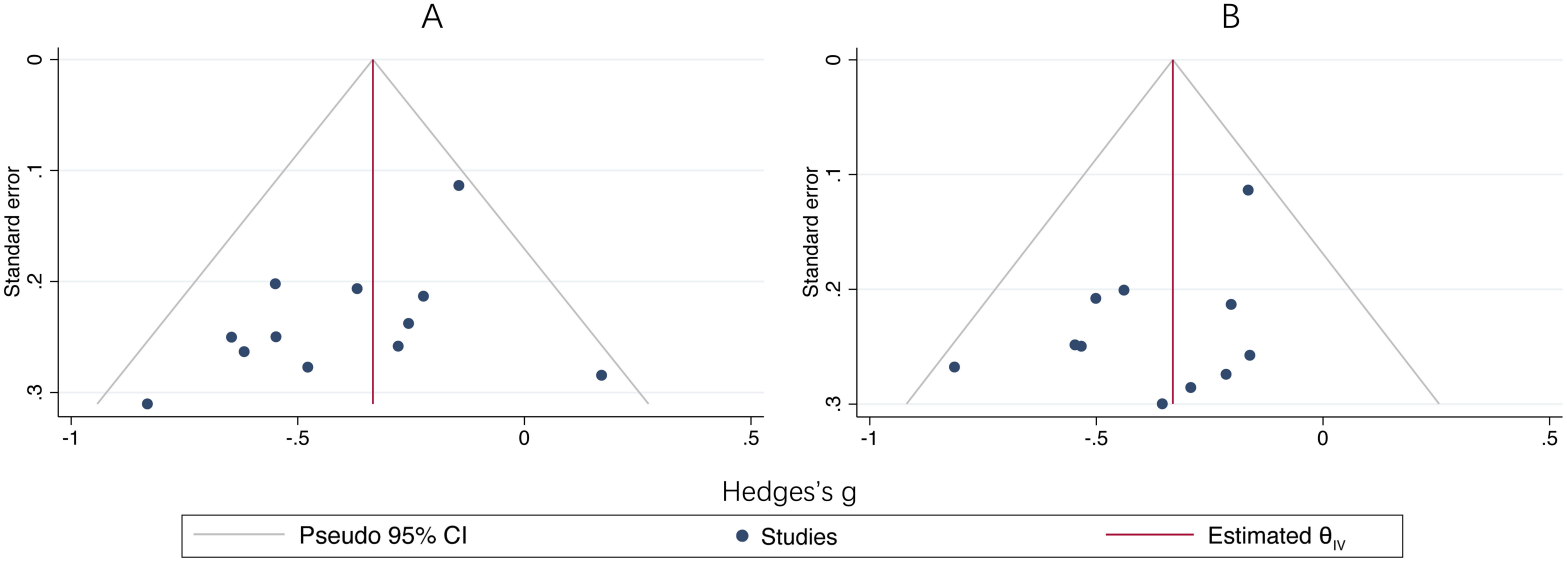
Results of Funnel plot. **(A)** Funnel plot for the outcome of pain scores, including 12 studies. **(B)** Funnel plot for the outcome of physical function, including 11 trials.

Funnel plots and Egger’s regression test were employed to explore potential publication bias for the primary outcomes. For pain scores (12 studies), visual inspection of the funnel plot (**Figure 5A**) showed a generally symmetrical distribution. While the Egger test did not reach standard statistical significance (*t* = -1.96, *p* = 0.078, **Table 6**), this result should be interpreted cautiously, as the test has limited statistical power when the number of included studies is small.

**Table 6:** Results of Egger’s test (Pain scores).

Std_Eff	Coefficient	Std. err.	t	P > |t|	[95% conf. interval]
slope bias	. 0442691	. 2040031	0.22	0.833	(-.4102781, -.4988164)
	-1.853624	.9454802	-1.96	0.078	(-3.960285, -.2530371)

Std. Err, standard error; t, *t*-test statistic; p, probability.

Similarly, for physical function (11 trials), the funnel plot (**Figure 5B**) appeared relatively symmetrical, and the Egger test was non-significant (*t* = -1.92, *p* = 0.087, **Table 7**). However, despite these non-significant test results, the possibility of publication bias cannot be definitively ruled out. Notably, the observed combination of uniform effect directions and a complete lack of statistical heterogeneity (*I^2^* = 0%) across the primary analyses may itself serve as a potential signal of unpublished negative findings. Therefore, while formal tests did not detect clear evidence of publication bias, the pooled estimates should still be interpreted with awareness of these methodological limitations.

**Table 7:** Results of Egger’s test (Physical function).

Std_Eff	Coefficient	Std. err.	t	P > |t|	[95% conf. interval]
slope bias	-.0214323	.1710462	-0.13	0.903	(-.4083656,.365501)
	-1.539753	.8010852	-1.92	0.087	(-3.351934,.2724275)

Std. Err, standard error; t, *t*-test statistic; p, probability.

### 3.8 Sensitivity analyses

**Figure 6:**
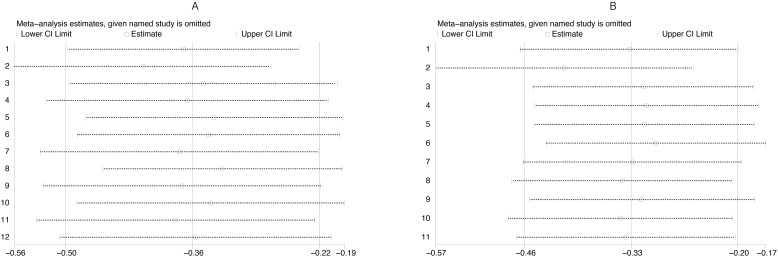
Results of sensitivity analyses. **(A)** Sensitivity analysis for the outcome of the pain scores. **(B)** Sensitivity analysis for the outcome of physical function.

Sensitivity analyses were conducted using a leave-one-out approach to assess whether the pooled estimates were influenced by any individual study. For both pain (**Figure 6A**) and physical function (**Figure 6B**), the omission of any single study did not materially change the pooled estimates. The direction and magnitude of the effects remained generally consistent across all iterations, suggesting that the main findings were not driven by any individual study.

## 4 Discussion

### 4.1 Main findings

The present study aimed to evaluate the efficacy of aquatic exercise on pain scores and physical function in patients with lower limb osteoarthritis, while exploring whether specific participant characteristics, exercise protocol parameters, or control group types influenced these clinical outcomes. To this end, we examined potential moderating factors, including participant characteristics, various training dosages, and the nature of the comparator groups.

Our meta-analysis demonstrated that the intervention yielded statistically significant, albeit small-to-moderate, reductions in pain and improvements in physical function compared with control conditions. Exploratory subgroup analyses suggested that favorable effects were observed across several categories. Notably, individuals categorized as obese showed a larger improvement in physical function than those who were overweight. However, no clear subgroup differences were observed according to intervention timing, volume, or comparator type. These findings suggest that participant characteristics may influence the magnitude of functional improvement, whereas current evidence does not support firm conclusions regarding optimal intervention parameters.

### 4.2 Clinical relevance

The clinical relevance of the observed effects should be interpreted cautiously. The pooled SMDs indicated small-to-moderate average effects on pain and physical function, suggesting that the expected clinical benefit may be modest at the group level. Although MCID thresholds are useful for interpreting scale-specific changes, direct comparison with these thresholds was limited in this review because the included studies used different instruments, including WOMAC, KOOS, NRS, BPI, and LAI, each with different scoring systems and MCID values. Therefore, the pooled SMDs provide standardized estimates of average treatment effects across different scales, but they do not allow a direct judgment of whether changes in each original scale exceeded its own MCID threshold. From a practical perspective, aquatic exercise may still be relevant as a low-impact component of conservative management, particularly for patients who have difficulty tolerating land-based exercise.

### 4.3 Pain scores

Our findings suggest that aquatic exercise was associated with lower pain scores in overweight or obese patients with lower limb OA. This finding is broadly consistent with previous meta-analyses. For example, Bartels et al. (2016) reported favorable effects of aquatic exercise on pain symptoms in patients with lower limb OA, and Xu et al. (2023) noted that pain improvement after aquatic exercise could be observed immediately after the intervention and may be maintained for up to three months. These findings support the potential role of aquatic exercise as a non-pharmacological option for pain management in lower limb OA.

In the subgroup analysis by participant characteristics, both overweight and obese participants appeared to experience reductions in pain scores, with a numerically larger effect observed in obese participants. This finding should be interpreted cautiously because the subgroup analyses were exploratory and the number of studies within each category was limited. One possible explanation is that individuals with higher body mass may derive greater benefit from buoyancy-related unloading during aquatic exercise, which can reduce weight-bearing stress on the knee and hip joints during movement (Thein and Brody, 1998; Becker, 2009; Psycharakis et al., 2022; Tedeschi et al., 2024). This explanation is also consistent with the mechanical and metabolic burden associated with excess body weight in lower limb OA (Sah et al., 1989; Torzilli et al., 1997; Egloff et al., 2012). Previous studies have suggested that aquatic exercise may be particularly relevant for overweight or obese patients with OA (Dias et al., 2017; Song and Oh, 2022). Nevertheless, the included trials did not directly assess joint loading, inflammatory markers, or neurophysiological pain modulation. Therefore, the potential biomechanical and neurophysiological explanations should be considered hypothesis-generating rather than direct mechanistic evidence.

Comparator type may also influence the interpretation of pain outcomes. In the subgroup analysis, the pain-related effect of aquatic exercise appeared clearer when compared with non-exercise comparators, whereas comparisons with exercise comparators were less precise and the confidence interval crossed the null value. However, the formal test for subgroup differences did not indicate clear evidence that comparator type modified the pain effect. Therefore, these findings should not be interpreted as evidence that aquatic exercise is superior to other exercise-based interventions. Instead, aquatic exercise may represent a useful low-impact exercise option, particularly when compared with non-exercise control conditions.

Exploratory subgroup analyses also suggested that programs with shorter session durations, lower weekly frequency, lower weekly volume, or shorter total intervention periods tended to show favorable pain outcomes. However, these patterns should not be interpreted as definitive evidence of an optimal dose-response relationship. Previous studies have discussed the possibility that exercise-induced hypoalgesia may depend on exercise dose, intensity, and duration, and that excessive exercise exposure may contribute to fatigue or discomfort in some patients (Wang et al., 2025; Janal et al., 1984; Koltyn, 2000; Hoffman et al., 2004; Naugle et al., 2012; Rice et al., 2019). In this context, shorter aquatic exercise sessions may be feasible for overweight or obese patients because they may provide an exercise stimulus while limiting fatigue-related pain fluctuation.

With respect to weekly exercise volume, the subgroup findings did not provide clear evidence that higher weekly duration produced greater pain reduction. Although some previous evidence has suggested that higher exercise volume may be beneficial for pain management (Haddadj et al., 2025), other studies have indicated that moderate weekly volumes may also be sufficient to achieve pain improvement (Wang et al., 2025; McCain, 1989; Hinman et al., 2007; Tomas-Carus et al., 2007; Munguía-Izquierdo and Legaz-Arrese, 2008). A possible explanation is that prolonged immersion or higher-volume aquatic exercise may increase fatigue or delayed-onset muscle soreness in some patients, which could influence pain perception or self-reported pain scores (Armstrong, 1984; Clarkson and Hubal, 2002; Ayán-Pérez et al., 2025). Nevertheless, this explanation was not directly tested in the included trials and should therefore be interpreted cautiously.

Similarly, the subgroup findings did not suggest a clear advantage of higher weekly frequency over lower weekly frequency for pain outcomes. This is consistent with previous evidence indicating that low- and high-frequency exercise interventions may not differ substantially in their effects on pain reduction (Husted et al., 2022). Trials using twice-weekly aquatic exercise have also reported pain improvement in patients with lower limb OA (Hinman et al., 2007; Rewald et al., 2020). For overweight or obese patients, lower-frequency aquatic exercise may be more feasible because this population may have reduced exercise tolerance and greater susceptibility to fatigue or pain fluctuation (Daenen et al., 2015; Zdziarski et al., 2015; Paley and Johnson, 2016).

The subgroup analysis also suggested that interventions lasting ≤ 8 weeks showed numerically greater pain reduction than those lasting >8 weeks. This observation may reflect early symptom improvement rather than the inherent superiority of shorter intervention protocols. Aquatic exercise may provide short-term pain relief through reduced weight-bearing, sensory stimulation, and improved movement confidence in water (Nathan and Rudge, 1974; McCain, 1986; Mannerkorpi et al., 2000; Busch et al., 2002; Melzack and Katz, 2004; Assis et al., 2006; Gowans and deHueck, 2007; Evcik et al., 2008). However, longer-term effects may be influenced by adherence, intervention monotony, and changes in participation over time (Chung et al., 2002; Cochrane et al., 2005; Smith et al., 2023; Shulman et al., 2025; Schleimer et al., 2026). Given the exploratory nature of these subgroup analyses, the current evidence does not allow definitive conclusions regarding the optimal intervention duration for pain reduction.

### 4.4 Physical function

This meta-analysis suggested that aquatic exercise was associated with better physical function in overweight and obese patients with lower limb OA compared with control conditions. Although the pooled estimate showed low statistical heterogeneity, clinical and methodological differences across the included studies should still be considered. Given the variations in comparator conditions, intervention duration, and exercise prescription parameters, the generalizability of these findings should be interpreted with caution.

Subgroup analyses suggested that participant characteristics may be related to the magnitude of functional improvement. Obese participants showed a numerically larger improvement in physical function than overweight participants. However, this finding should be interpreted cautiously because the subgroup analyses were exploratory and the number of studies within each category was limited. For overweight and obese patients, excess body weight is an important risk factor for lower limb OA and may increase mechanical loading during land-based functional activities (Raud et al., 2020; Sampath et al., 2023; Shumnalieva et al., 2023). The aquatic environment may reduce weight-bearing stress and allow patients to perform functional movements with a greater range of motion and less joint loading (Abadi et al., 2019; Oral et al., 2021). In addition, water resistance may provide a low-impact training stimulus during movement. Because obese patients often have larger limb dimensions and body surface areas, they may experience greater water resistance during similar movements (Ma et al., 2021), which could partly contribute to functional improvement without the need for additional external loading (Pöyhönen et al., 2000; Torres-Ronda and Del Alcázar, 2014). Nevertheless, these explanations remain hypothetical because the included trials did not directly assess joint loading, muscle activation, or biomechanical adaptation.

Regarding comparator type, the subgroup analysis suggested that the effect of aquatic exercise on physical function appeared clearer when compared with non-exercise comparators, whereas comparisons with exercise comparators were less precise and did not clearly favor aquatic exercise. However, the formal test for subgroup differences did not provide clear evidence that comparator type modified the functional effect. Therefore, these findings should not be interpreted as definitive evidence that aquatic exercise is superior to other exercise-based interventions. Rather, aquatic exercise may represent a useful low-impact exercise option, particularly when compared with non-exercise control conditions.

The subgroup analyses showed no clear between-group differences in functional improvement according to weekly intervention frequency, single-session duration, or total weekly intervention duration. Lower-dose regimens, such as sessions lasting < 45 minutes and weekly exercise volumes of ≤ 120 minutes, showed numerically favorable effect estimates. These findings suggest that modest aquatic exercise doses may be sufficient to elicit functional gains in some overweight or obese patients with lower limb OA, although they should not be interpreted as evidence of an optimal dose-response relationship. Previous studies have suggested that functional improvement after exercise may be related to enhanced movement confidence, neuromuscular control, balance regulation, and task-specific training adaptations (Whitfield et al., 2025; Ditroilo et al., 2018; Conceição et al., 2022; Gao et al., 2024). In the aquatic environment, reduced weight-bearing stress and multidirectional water resistance may allow patients to practice functional movements with less joint loading while still receiving an appropriate training stimulus (Roddy et al., 2005; Gabriel et al., 2006; Hoops et al., 2012; Bove et al., 2017; Wang et al., 2024). Therefore, lower-volume aquatic exercise protocols may be a feasible and clinically practical option for this population, particularly for patients with limited exercise tolerance.

The subgroup analysis suggested that interventions lasting ≤ 8 weeks showed numerically larger functional improvements than those lasting > 8 weeks. This pattern may reflect early functional adaptation rather than the inherent superiority of shorter intervention protocols. In the early phase of aquatic exercise, reduced weight-bearing stress, hydrostatic pressure, and repeated movement practice in a supportive environment may help improve joint mobility, reduce perceived stiffness, and enhance confidence in functional movement (Bender et al., 2005; Gabay et al., 2008; Becker, 2009; Fioravanti et al., 2010). These factors may partly explain the early functional gains observed in some studies. In addition, longer-term intervention effects may be influenced by adherence, intervention monotony, and participant engagement over time (Bartels et al., 2016; Song and Oh, 2022; Schleimer et al., 2026). Therefore, the apparent advantage of shorter intervention periods should be interpreted as exploratory rather than as evidence that interventions lasting ≤ 8 weeks are optimal for improving physical function.

### 4.5 Limitations

A primary limitation relates to the relatively modest number of included trials and the associated total sample size. Although this review included 12 studies involving 1,057 participants, the number of studies available for several subgroup analyses was limited, which may have reduced the statistical power to detect more nuanced differences across BMI categories, exercise prescription parameters, and comparator types. Therefore, these subgroup findings should be interpreted as exploratory and hypothesis-generating rather than confirmatory evidence of subgroup effects or dose-response relationships.

Second, although statistical heterogeneity was low, there was substantial clinical and methodological heterogeneity across studies, including differences in aquatic exercise modality, disease site, comparator condition, intervention duration, exercise intensity, water temperature, supervision, and adherence reporting. These differences may limit the generalizability of the pooled estimates.

Third, comparator conditions varied across studies. Although subgroup analyses by comparator type were conducted, the limited number of studies using exercise comparators means that the comparative efficacy of aquatic exercise relative to other exercise-based interventions remains uncertain.

Finally, the use of standardized mean differences across different outcome instruments limited our ability to determine whether the observed pooled effects exceeded scale-specific MCID thresholds. Therefore, statistical improvements should not be directly equated with clinically meaningful improvements for all patients.

## 5 Conclusions

Aquatic exercise may be associated with modest improvements in pain scores and physical function in overweight or obese patients with lower limb OA. Exploratory subgroup analyses suggested that individuals categorized as obese showed numerically larger improvements than those categorized as overweight. In addition, lower-volume protocols, including lower weekly frequency, shorter intervention duration, and shorter session length, appeared to be associated with favorable outcomes. However, these findings should be interpreted cautiously because the subgroup analyses were exploratory, the number of studies within several categories was limited, and the certainty of evidence was low. Therefore, the current evidence does not allow firm conclusions regarding the optimal aquatic exercise prescription or a definitive dose-response relationship. Aquatic exercise may represent a useful low-impact adjunct to conservative management, particularly for overweight or obese patients who have difficulty tolerating land-based exercise. Further well-designed trials are needed to confirm clinically meaningful effects and to clarify the optimal dose, comparator-specific effects, and long-term effectiveness of aquatic exercise in this population.

## Data Availability

The original contributions presented in the study are included in the article/**Supplementary Material**. Further inquiries can be directed to the corresponding authors.
