# Supplementary material for: Effects of aquatic exercise on pain and physical function in overweight/obese patients with lower limb osteoarthritis: a systematic review and meta-analysis
Source: Front Physiol. 2026 Jun 16;17:1864401. doi: 10.3389/fphys.2026.1864401 (PMC13321089; doi:10.3389/fphys.2026.1864401)
Supplement: Supplementary file 1 [file DataSheet1.pdf]

Supplementary Material

Table S1 Full electronic search strategies for all databases.

Database	Complete Search Strategy	Results
PubMed	("Osteoarthritis"[Mesh] OR "Osteoarthritis"[Title/Abstract] OR "Osteoarthrosis"[Title/Abstract] OR "Degenerative Joint Disease"[Title/Abstract]) AND ("Aquatic Therapy"[Mesh] OR "Hydrotherapy"[Mesh] OR "Swimming"[Mesh] OR "Aquatic exercise"[Title/Abstract] OR "Water-based exercise"[Title/Abstract] OR "Pool exercise"[Title/Abstract] OR "Underwater exercise"[Title/Abstract])	365
Web of Science	(TS=("Osteoarthritis" OR "Osteoarthrosis" OR "Degenerative Joint Disease")) AND (TS=("Aquatic Therapy" OR "Hydrotherapy" OR "Swimming" OR "Aquatic exercise" OR "Water-based exercise" OR "Pool exercise" OR "Underwater exercise"))	338
Embase	('osteoarthritis'/exp OR 'osteoarthritis' OR 'osteoarthritis':ti,ab OR 'osteoarthrosis':ti,ab OR 'degenerative joint disease':ti,ab) AND ('aquatic therapy'/exp OR 'hydrotherapy'/exp OR 'swimming'/exp OR 'aquatic exercise':ti,ab OR 'water-based exercise':ti,ab OR 'pool exercise':ti,ab OR 'underwater exercise':ti,ab)	752
Cochrane Library	([mh "Osteoarthritis"] OR ("Osteoarthritis"):ti,ab,kw OR ("Osteoarthrosis"):ti,ab,kw OR ("Degenerative Joint Disease"):ti, ab, kw) AND ([mh "Aquatic Therapy"] OR [mh "Hydrotherapy"] OR [mh "Swimming"] OR ("Aquatic exercise"):ti, ab, kw OR ("Water-based exercise"):ti,ab,kw OR ("Pool exercise"):ti, ab,kw OR ("Underwater exercise"):ti,ab,kw)	147
Scopus	TITLE-ABS-KEY ("Osteoarthritis" OR "Osteoarthrosis" OR "Degenerative Joint Disease") AND TITLE-ABS-KEY ("Aquatic Therapy" OR "Hydrotherapy" OR "Swimming" OR "Aquatic exercise" OR "Water-based exercise" OR "Pool exercise" OR "Underwater exercise")	754

Table S2. Assessment and reporting of adverse events across included trials

Study	Adverse event assessment/reporting	Summary of adverse events
Alkatan 2016	Assessed, but incompletely reported	Int: chlorine sensitivity, n=1; unrelated injury, n=1. Con: knee pain during cycling, n=2.
Cochrane 2005	Assessed, but incompletely reported	Int: minor bruising after slipping near pool/changing room, n=2.
Dias 2017	Assessed, but incompletely reported	No treatment-related harm reported.
Fransen 2007	Assessed and reported	Serious adverse events requiring hospitalization, n=11; none intervention related. Int: withdrawal due to low back pain exacerbation, n=1.
Hinman 2007	Assessed and reported	Minor transient events. Int: mild joint discomfort, n=17; mild lumbar pain, n=3; calf/foot cramps, n=2.
Kunduracilar 2018	NR	NR
Kuptniratsaikul 2019	Assessed and reported	Int: joint pain, n=3; muscle pain, n=4; other, n=1. Con: joint pain, n=4; muscle pain, n=7; other, n=5. No between-group difference.
Lim 2010	Assessed and reported	Int: dropout due to heart/chest pain, n=1, not arthritis related.<br>Con: dropout due to pain/discomfort, n=3.
Regazzo 2026	NR	NR
Rewald 2020	Assessed and reported	Int: hyperventilation at end of training, n=1; hospitalized overnight and resumed training after rest. Increased knee pain after training, n=10; symptom exacerbation after strength testing, n=4.<br>Con: no adverse events.
Waller 2017	Assessed and reported	Int: stopped due to pain after resistance boots, n=1; dyspnoea but completed after education, n=1.
Wang 2011	Assessed and reported	Int: dizziness during exercise, n=1.

Abbreviations: Int = intervention group receiving aquatic exercise; Con = comparator/control group; NR = not reported.
